# Characterization and clustering of intra-day physical activity patterns using accelerometry among sexual and gender minority adults

**DOI:** 10.1186/s12889-025-23425-5

**Published:** 2025-07-03

**Authors:** David Lopez-Veneros, Billy A. Caceres, Kasey Jackman, Joseph A. Belloir, Yashika Sharma, Suzanne Bakken, Ipek Ensari

**Affiliations:** 1 Columbia University School of Nursing, New York, NY 10032 USA; 2 The Data Science Institute at Columbia University, New York, NY 10027 USA; 3 University of Connecticut, Storrs, CT 06269 USA; 4 Department of Artificial Intelligence and Human Health, Icahn School of Medicine at Mount Sinai, Hasso Plattner Institute for Digital Health at Mount Sinai, Blavatnik Family Women’s Health Research Institute, New York, NY 10029 USA

**Keywords:** Physical activity, Accelerometry, Sexual and gender minority adults, Phenotyping, Unsupervised learning

## Abstract

**Background:**

Sexual and gender minority (SGM) adults experience significant health disparities linked to chronic exposure to minority stressors (e.g., discrimination), and could be reciprocally associated with physical activity (PA) behavior. While PA is a health-protective factor, research on PA patterns in SGM adults is limited. Identifying potential latent PA profiles can inform tailored behavior change approaches.

**Objective:**

To investigate latent profiles (i.e., clusters) of daily PA trajectories among sexual and gender minority (SGM; lesbian, gay, bisexual, transgender, queer) adults using functional latent block models (FLBMs), a co-clustering technique that simultaneously accounts for variation at the individual- and day-level.

**Study sample:**

The study included 42 Black and Latinx SGM adults who wore Fitbit trackers for up to 30 days of PA data collection as a part of a sleep health study, yielding 1,209 person-level days of step count data.

**Methods:**

Each 24-h period of step counts was smoothed using Fourier-transform to create the functional data matrix and fit the FLBMs. The optimal number of clusters was determined using the integrated completed likelihood (ICL) criterion.

**Results:**

The best-fitting model identified 3 individual-level clusters (*K*) based on the daily step count patterns (ICL = -88,495.88). *Low activity* cluster (*n* = 11) was characterized with the lowest overall PA, slightly later bedtimes, and the least intra-day and hourly variability. *Steady moderate activity* cluster (*n* = 23) was characterized by a gradual increase in step counts that spread over the course of the day, with a small peak in the afternoon. *Fluctuating high activity* cluster was characterized by a peak in activity earlier in the day, compared to other clusters. Cluster 3 membership was also associated with the highest volume of PA overall, along with hourly and daily variability in step counts and higher intensities of PA. The model secondarily identified 2 day-level clusters (*L*), representing weekday and weekend PA patterns.

**Conclusions:**

We identified distinct habitual daily PA trajectories among SGM adults based on daily volume and variability. Analyzing individual PA variances can help identify inactive periods and individuals at higher risk, which can inform the design of tailored interventions and self-management strategies to promote PA.

**Supplementary Information:**

The online version contains supplementary material available at 10.1186/s12889-025-23425-5.

## Background

Despite the established benefits of physical activity (PA) on mental and physical health, the global prevalence of physical inactivity in adults has increased in the last two decades [1]. While racial and ethnic disparities in PA behaviors among adults have been investigated, research on PA patterns among sexual and gender minority (SGM; e.g., lesbian, gay, bisexual, transgender, queer) individuals remains limited. According to the minority stress model [2, 3], SGM individuals experience mental and physical health challenges as a result of chronic exposure to gender minority stressors, such as experiences of discrimination, expectations of rejection, and concealment of one’s SGM identity. SGM individuals experience a substantially higher prevalence of physical and mental health problems compared to their heterosexual and cisgender (i.e., non-transgender) counterparts [4]. These disparities include higher likelihood of having cardiovascular disease, certain types of cancer, HIV, anxiety, depression, substance use disorders, and suicidality [5–9]. Although PA can serve as a significant factor against these health disparities, our understanding of PA patterns among SGM individuals is limited and inconsistent. Some studies indicate that SGM individuals report greater PA than non-SGM individuals [10–12]. In contrast, other studies using self-reported PA data indicate that SGM people are less likely to be physically active and more likely to be sedentary than non-SGM people [13–17]. Qualitative research has identified specific barriers that may explain these PA disparities, finding that experiences of discrimination, exclusion, or fear of being discriminated against in PA settings deter SGM individuals from obtaining adequate PA [18–21]. Given the mixed findings on PA patterns among SGM adults, coupled with established health disparities, investigation of whether distinct SGM sub-groups based on PA patterns exist can be a crucial starting point toward elucidating the specific barriers, incentives, and preferences that influence PA and sedentary behaviors in this population.

The discrepancy across PA findings among SGM individuals could partly be explained by the different PA measurement techniques. Existing studies have largely relied on self-reported PA (i.e., questionnaires), which is susceptible to potential inaccuracies due to response and recall bias [22–24]. While a few studies report pedometer-based step counts among sexual minority women [25, 26], there have been no studies to date reporting accelerometer-based PA estimates among SGM adults. This gap in the literature further supports the need for research that uses accelerometry-based, objectively-estimated methods to characterize PA patterns in SGM adults.

Beyond objectivity, the continuous, longitudinal, and granular information accelerometry provides regarding activity patterns enables greater insight into between- and within-individual variances. When coupled with appropriate data analytic methods, these features can be particularly useful for investigating possible distinct, latent profiles or clusters ("phenotypes") of PA patterns. Investigation of possible phenotypes that exist within the SGM population is relevant given the mixed results from previous research and heterogeneity in the needs, preferences and other individual-level characteristics that influence PA behavior among SGM adults. There is also growing evidence suggesting that spreading PA across the day may yield better health outcomes compared to concentrating PA within specific time slots while spending extended periods of time sitting or lying down [27–29]. Accordingly, this investigation can help identify individuals or periods susceptible to physical inactivity, as well as the distinct PA patterns that otherwise would be challenging to uncover among diverse populations with variable habitual activity patterns using aggregated data.

The objective of this study was to investigate potential phenotypes of accelerometry-based daily PA trajectories among SGM individuals via functional latent block models (FLBMs) [30]. FLBM is a co-clustering technique that relies on mixture modeling of hierarchical data and accommodates multivariate time-dependent data to simultaneously account for subject heterogeneity and dependency between time points (i.e., days). We hypothesized that there would be multiple homogenous subgroups (i.e., clusters) of SGM individuals based on differences in the intraday step count trends. We secondarily hypothesized that the model-estimated clusters would further differ with respect to other PA parameters including minutes spent in different intensities.

## Methods

### Study sample

The study included 42 SGM adults recruited for a daily diary study that aimed to investigate sleep health and related outcomes among Black and Latinx SGM adults using 30 days of ecological momentary assessments (EMAs). Eligibility criteria included identification as both an SGM person and a person of color, 18+ years of age, residing in the United States (U.S.), and owning a smartphone device. Those with diagnosed sleep disorders (e.g., insomnia, obstructive sleep apnea), major co-morbidities (e.g., cardiovascular disease) and any evidence of cognitive impairment based on a six-item cognitive screener [31] were excluded. This screener is a brief and reliable instrument for identifying subjects with cognitive impairment and its diagnostic properties are comparable to the full mini-mental state examination (MMSE) [31]. Participants were recruited from April 2021 to April 2022 through advertisements on Facebook, Twitter, and ResearchMatch [32, 33], and included a link to an online screening form on REDCap [33, 34]. ResearchMatch is a nonprofit program funded by the National Institutes of Health with a large population of volunteers who have consented to be contacted by researchers about health studies.

### Data collection

The Institutional Review Board at the Columbia University Irving Medical Center provided ethical approval for the study. Data collection procedures for the parent study are described in detail elsewhere [35]. Briefly, participants completed three virtual visits via Zoom. During Visit 1 (45–60 min), participants provided electronic informed consent via REDCap. Wrist-worn activity trackers (Fitbit Inspire 2) were then mailed to participants for the 30-day PA data collection. Approximately 1 week later at Visit 2 (~ 30 min), participants received detailed instructions regarding the use of the Fitbit and the study protocol. Following 30 days of EMAs and Fitbit tracking, participants completed Visit 3 (~ 30 min) which involved completing an exit survey with questions on their usual sleep habits during the past month and acceptability of the study protocol. Participants were compensated $100 for completing all study components.

### Outcome variables

The Fitbit trackers were used for estimates of step counts, time spent in different intensities (e.g., low, moderate, high) of PA, and sedentary behavior. Raw Fitbit data were processed and validated based on Fitbit’s propriety algorithms. Fitbit uses its own proprietary algorithm to determine wear time based on heart rate and activity data [36]. However, this method can still leave days with likely non-wear time (e.g., zero activity counts during the entire awake period). Therefore, to ensure data quality, we used 12 or more hours of non-zero counts to determine adequate wear time and classify as a valid day. All participants with at least three days of data based on this criterion were included in the analysis, leading to removal of one participant. Similar approaches have been used by other studies, as well as more flexible cut-off periods (e.g., eight hours of wear time), including work by our team [37–40].

We used the step count variables as the outcome for clustering, motivated by the following factors. First, the study hypothesis focused on overall daily activity patterns, which includes all intensities and types. Second, step counts data tend to fluctuate more often than other PA parameters, therefore can better capture group differences based on these nuances in the data trends. Third, from a modeling stance, zero-inflated data matrices (e.g., days with high amounts of sedentary minutes) pose the risk of biased and unstable model estimates [41]. Finally, there are published guidelines and research-based cut-off points for daily step counts linked to health outcomes [42–45], which allows interpretation of the results in their context, therefore making the identified clusters more interpretable. These collectively provided motivation to use step counts as the primary model outcome.

To provide a comprehensive description of the model-estimated clusters and make comparisons afterwards, we calculated summary scores for low-, moderate- and vigorous intensity PA (i.e., variables not used for the clustering algorithm).

### Data processing

Missing data (11.8%) were imputed using multivariate imputation by chained equations (MICE) with predictive mean matching (PMM) using the R mice package [46]. Fifteen imputed datasets were generated following published guidelines on this method [46–48]. PMM computes predictions based on the assumption that the missing values follow the distributional characteristics of the observed data. This prevents non-plausible imputations (e.g., negative step counts) and values outside the observed data range. PMM is considered versatile and robust against model misspecification, and performs well (i.e., least biased estimates) under a variety of scenarios [49, 50]. Imputation quality was assessed using graphical diagnostics (density and convergence plots) and statistical indices including fraction of missing information (FMI) and relative information variance (RIV) [51].

### Functional clustering analysis

Functional latent block models (FLBMs) were used for clustering analyses. FLBM is a co-clustering technique designed for hierarchical and multivariate time-dependent data. It accommodates within-subject dependence across multiple days without aggregating or losing temporal information. Specifically, FLBM clusters entire smoothed daily trajectories rather than individual hourly data points. This preserves meaningful temporal patterns in PA throughout each day. Accordingly, each participant’s data are represented by 30 sample paths (i.e., 30 days with 24 h each). To accomplish this, discrete hourly step-count data points are transformed into continuous smoothed trajectories ("functional" form) using Fourier basis functions. We selected Fourier smoothing due to its suitability for periodic data such as daily activity patterns. The model simultaneously identifies clusters at two dimensions: individual-level clusters representing distinct PA patterns among participants ("rows"), and day-level clusters representing differences in PA patterns across days ("columns").

### Clustering model selection and inference

Model inference was conducted using a stochastic expectation–maximization Gibbs sampling algorithm [30, 52]. While clustering in general is an exploratory technique, clustering models intrinsically assume and test the existence of unobserved heterogeneity within the data that can be separated into homogeneous subgroups [53–55]. The optimal number of individual-level clusters (*K*) and day-level clusters (*L*) was determined based on the integrated completed likelihood criterion (ICL). The ICL is estimated based on the Bayesian information criterion (BIC) and includes an additional term (estimated mean entropy). ICL penalized overlapping or indistinct clusters, ensuring clear separation between identified groups (i.e., indicating low distinction between clusters, and is well-suited for model-based clustering tasks) [30, 56–58]. Cluster validity was further assessed visually by examining separation between cluster trajectories. (i.e., maximal distance between different clusters, minimal distance between points of the same cluster).

### Characterization of clusters

To characterize the model-identified clusters based on person-level factors, linear mixed-effects regression models were conducted to compare clusters on total daily minutes spent in different PA intensities (light, moderate, vigorous). Significance tests for fixed effects parameters were conducted using t-tests with degrees of freedom approximated by Satterthwaite's method. Demographic differences across clusters were assessed using chi-squared (*X*^*2*^) tests for categorical variables (i.e., sex assigned at birth, gender, sexual orientation, race, ethnicity, education levels, household income, and employment status) and linear regression for continuous variables (age). Finally, to contextualize the study sample, we assessed scores on self-reported sleep disturbance (PROMIS sleep disturbance scale) [59], insomnia (Insomnia Severity Index; ISI) [60], and sleep quality (Pittsburgh Sleep Quality Index; PSQI) [61] for those who had available data on these outcomes (*N* = 33). A global score of > 5 on the PSQI has been suggested to distinguish "poor sleepers" from "good sleepers," with sensitivity of 89.6% and specificity of 86.5% [61]. Similarly, the ISI cut-off scores of 8 and 15 suggest subthreshold and clinical insomnia [60]. Significance level for all statistical tests was set at *p* < 0.05.

## Results

### Sample characteristics

Participants (*N* = 42) provided 1,209 person-level days of accelerometry data in total. Sample characteristics are provided in Table 1. The mean age was 27.1 years (SD = 7.73, Range = 18–57), and 85.7% (*n* = 36) of the participants self-identified as Latinx (regardless of race).

**Table 1:** Sample characteristics

Demographic Characteristics	Mean (SD)/*N* (%)
Age (in years, range 18–57)	27.1 (7.73) (*Median* = *26*)
Female Sex at Birth	28 (66.7)
Gender	
Cisgender	28 (66.7)
Genderqueer or genderfluid	6 (14.3)
Non-binary	5 (11.9)
Transgender	3 (7.1)
Sexual Identity	
Lesbian/gay	16 (38.1)
Bisexual	16 (38.1)
Queer	7 (16.7)
Pansexual	2 (4.7)
Straight/heterosexual	1 (2.4)
Race	
Black or African American	4 (9.5)
Native Indian or Alaska Native	2 (4.8)
Multi-racial	19 (45.2)
White	17 (40.5)
Latinx Ethnicity	36 (85.7)
BMI	
18.5–24.9 (Normal weight)	20 (47.6)
25–29.9 (Overweight)	9 (21.5)
30–34.9 (Obese Class I)	8 (19.1)
35–39.9 (Obese Class II)	2 (4.7)
> 40 (Obese Class III)	3 (7.1)
Employment	
Working full-time	22 (52.4)
Working part-time	13 (31.0)
Retired or disabled	1 (2.4)
Not looking for work, for other reasons	3 (7.1)
Working multiple jobs	3 (7.1)
Annual Income	
< 22.5 K	10 (23.8)
22.5 K—39.999 K	10 (23.8)
40 K—74.999 K	12 (28.6)
> 75 K	10 (23.8)
Physical Activity	
1 Day a week	7 (16.7)
2 Day a week	8 (19.1)
3 Day a week	10 (23.8)
4 Day a week	3 (7.1)
5 Day a week	6 (14.3)
6 Day a week	1 (2.3)
7 Day a week	7 (16.7)

### FLBM-based estimation of clusters

**Fig. 1:**
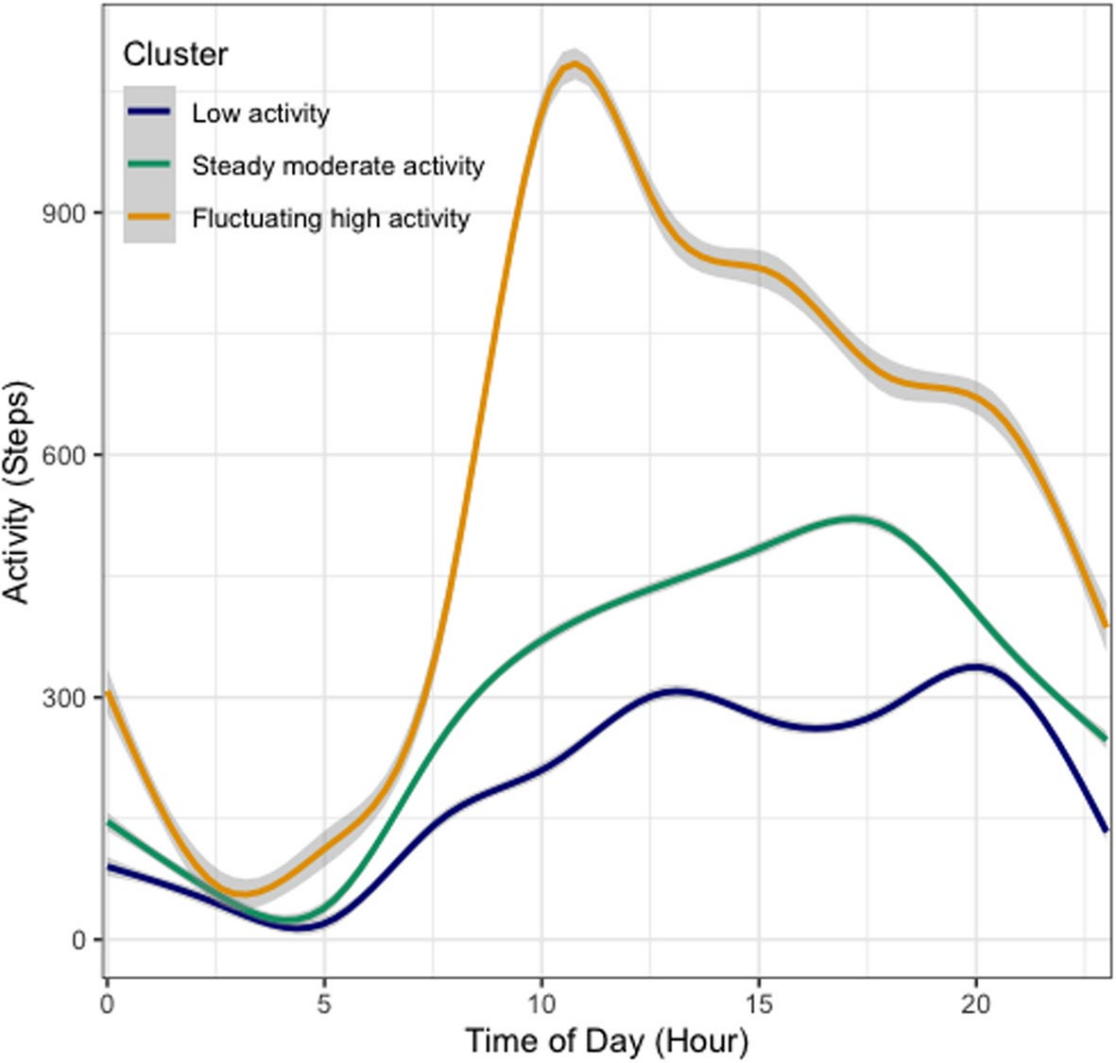
Latent clusters of PA patterns at the individual level. 3 distinct clusters of PA patterns at the individual level were identified: (1) *Low activity* cluster (representing the lowest overall activity distributed throughout the day in a relatively even manner, and a minor dip coinciding with the earlier afternoon hours of the day), (2) *Steady moderate activity* cluster (with a steady PA distribution, little variability throughout the day with lower activity levels during the earlier hours and a small peak in the afternoon), (3) *Fluctuating high activity* cluster (representing the most active participants, with earlier start in daily activity, higher activity in the earlier hours, and remaining active during the evening hours)

We selected the final FLBM yielding the highest ICL value based on the pooled results from the models run on each imputed dataset (*N* = 15, with 100 iterations of the SEM-Gibbs algorithm per model). The best-fitting model (ICL_pooled_ = −88,495.88) estimated 3 individual-level clusters (*K*) and 2 day-level clusters (*L*) based on differences in both the distribution and total amount of hourly PA over the course of the day. A visual depiction of the intraday PA trajectories of the model-estimated clusters is provided in Fig. 1.

### Characterization of individual- and day-level clusters

**Fig. 2:**
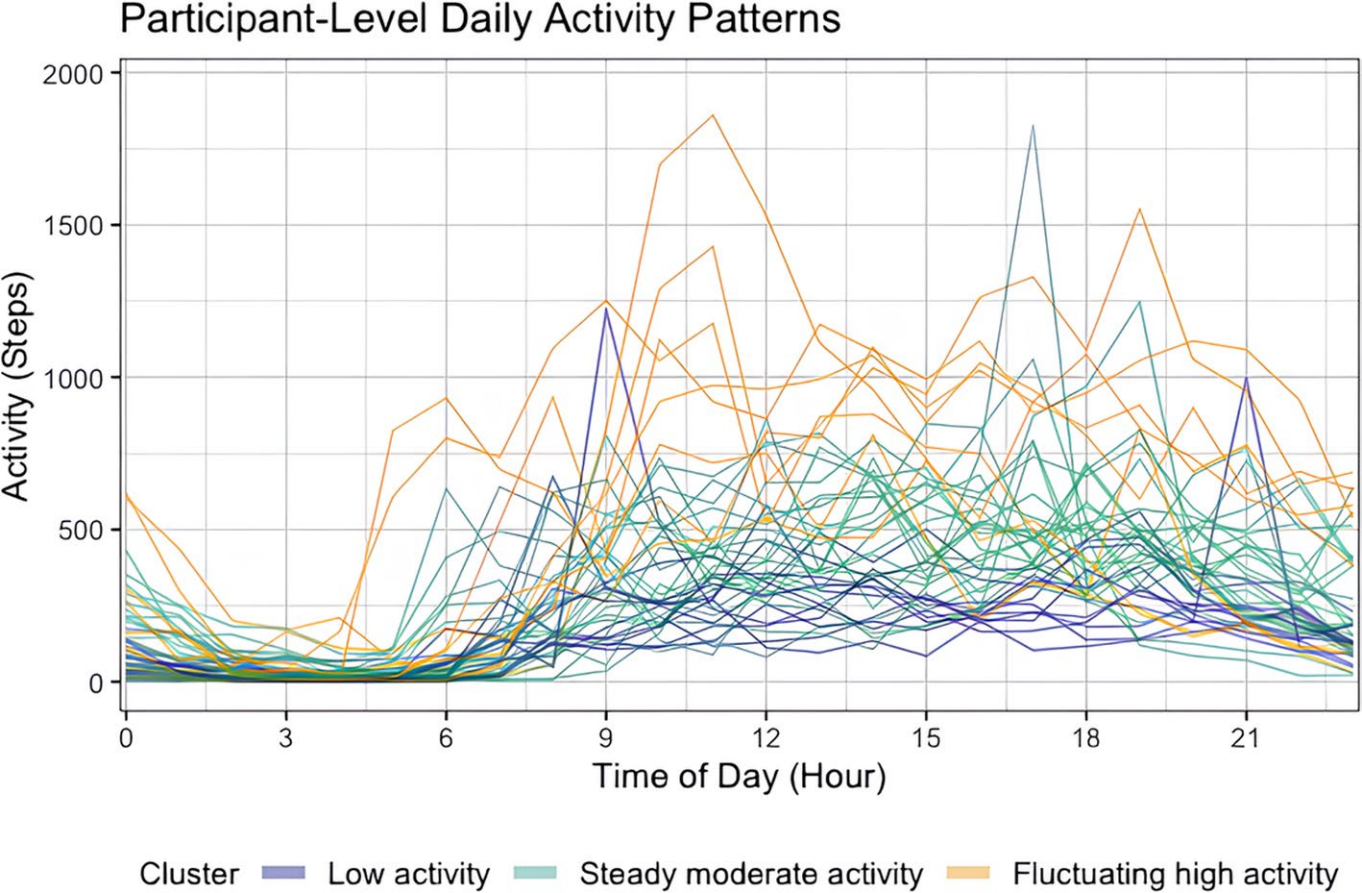
Observed person-level mean daily step counts over the course of 24 h (*N* = 42). Each line represents an individual participant's daily activity trajectory, with colors indicating their cluster membership: Low activity (navy blue), Steady moderate activity (green), and Fluctuating high activity (yellow)

Cluster-level descriptive statistics on all PA parameters are provided in Table 2. Figure 2 depicts the observed means of each individual’s daily step counts. Based on our analysis, we refer to the three identified clusters as *‘Low activity’*,* ‘Steady moderate activity’,* and* ‘Fluctuating high activity’*. The first cluster—*‘Low activity’* (*n* = 10)—was characterized by the lowest overall step counts distributed throughout the day in a relatively consistent manner. The trajectory for this cluster indicated a minor dip that coincided with the early afternoon hours of the day (See Fig. 1). Membership in the *Low activity* cluster was similarly associated with the lowest volume of total PA (i.e., minutes of low-, moderate- and high intensity; see Tables 2 and 3), and the least variability in hourly step counts based on the point estimates of the random intercept (i.e., B_1_ = 287.86, B_2_ = 466.93, B_3_ = 762.071, respectively, *p* < 0.0001). The second cluster – ‘*Steady moderate activity’*—comprised the largest group (*n* = 23) and the PA pattern was characterized by a gradual increase in step counts that spread over the course of the day (See Fig. 1). Participants in this cluster accumulated 34 min of total moderate-to-vigorous PA (MVPA) on average (See Table 2). Of note, membership to the *Steady moderate activity* cluster was associated with lower activity levels during the earlier hours of the day (vs. later hours), with a small peak in the afternoon. The third cluster—*‘Fluctuating high activity’*—was the smallest cluster (*n* = 8) and was characterized by the highest amount of overall step counts and a distinct peak in activity earlier in the day compared to other clusters (See Fig. 1). Membership to this cluster was also associated with an earlier start of daily activity. Tapering of daily activity in the evening was similar in terms of timing, but steeper, indicating greater likelihood of maintaining activity during evening hours. Finally, the *Fluctuating high activity* cluster had the highest average total MVPA (Mean = 74.98 min; See Table 2), as well as the highest within-individual (i.e., day-to-day) variability in MVPA compared to other clusters, based on the analysis of variance (F_(2,39)_ = 4.53, *p* = 0.016).

**Table 2:** Descriptive statistics for cluster-level daily total minutes of PA in each intensity and step counts (*N* = 42)

	**Cluster 1** ***Low activity* (*n* = 11)**		**Cluster 2** ***Steady moderate activity* (*n* = 23)**		**Cluster 3** ***Fluctuating high activity* (*n* = 8)**	
**PA parameter**^a^	**Mean (SD)**	**Range** **(Min–Max)**	**Mean (SD)**	**Range** **(Min–Max)**	**Mean (SD)**	**Range** **(Min–Max)**
Light PA (mins)	163.03 (69.32)	0–394	202.28 (98.18)	0–639	246.08 (119.61)	0–590
Moderate PA (mins)	12.28 (243.51)	0–217	15.362 (23.23)	0–269	38.07 (41.18)	0–238
Vigorous PA (mins)	10.17 (23.88)	0–223	18.12 (28.12)	0–282	36.90 (42.42)	0–337
Steps counts	4695 (367)	2,868–6,461	7115 (732)	4,712–10,400	13,349 (894)	10,384–17,895

^a^Based on imputed data

**Table 3:** Results from linear mixed-effects regression models comparing physical activity intensities across clusters

**PA Variable** (minutes)	**Participant Cluster**	**Point Estimate (SE)**	**df**	**t statistic**	**Adjusted *p*-value**
Light intensity PA	Low activity	162.88 (17.22)	39.99	9.45	< 0.0001
	Steady moderate activity	39.62 (20.96)	39.09	1.89	0.066
	Fluctuating high activity	83.20 (26.53)	38.89	3.13	0.003
Moderate intensity PA	Low activity	12.25 (2.96)	37.98	4.13	0.0001
	Steady moderate activity	3.74 (3.61)	38.27	1.03	0.307
	Fluctuating high activity	25.82 (4.56)	37.66	5.66	< 0.0001
Vigorous intensity PA	Low activity	10.13 (3.44)	38.77	2.93	0.005
	Steady moderate activity	8.12 (4.20)	39.03	1.93	0.060
	Fluctuating high activity	26.77 (5.30)	38.48	5.04	< 0.0001

**Fig. 3:**
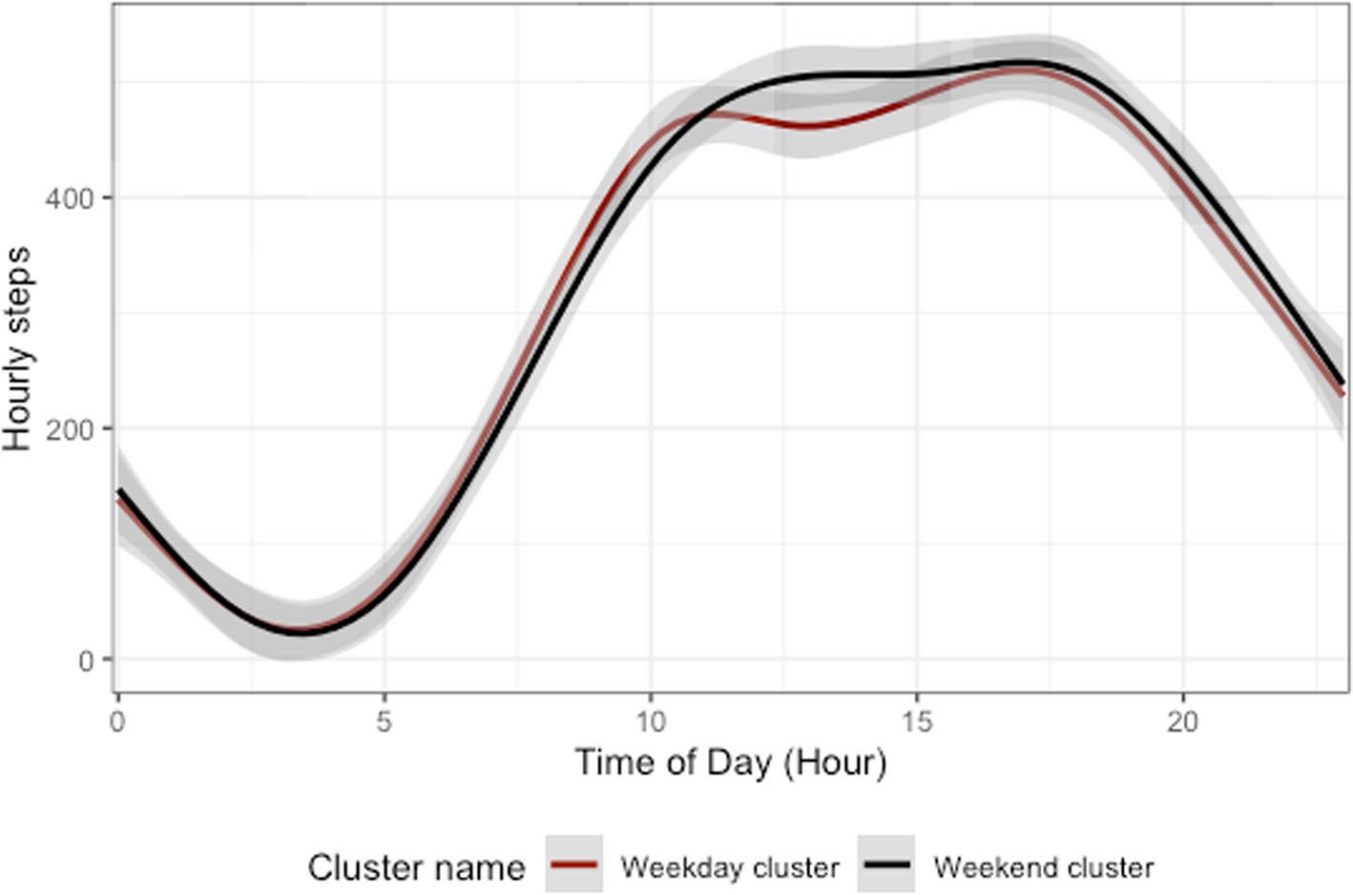
Latent clusters of PA patterns at the day level. Day-level (“*Weekday”* and *“Weekend”*) clusters identified from the FLBM co-clustering of the column-level data (*N* = 30 distinct days)

As a secondary outcome, the model identified two clusters based on the day- (i.e., column) level data independent of individual-level data. Figure 3 provides a visual depiction of these trajectories, which could be referred to as *“Weekday”* and *“Weekend”* clusters based on the post-hoc *X*^*2*^ tests for pairwise comparisons (overall test *X*^*2*^ = 708.39, *df* = 6, *p* < 0.0001). The *Weekday* cluster was significantly more likely to include Tuesdays (7.064, *p* < 0.0001), Wednesdays (15.144, *p* < 0.0001), and Thursdays (11.682, *p* < 0.0001), whereas the *Weekend* cluster was more likely to be representative of Saturdays (10.313, *p* < 0.001), and Sundays (16.293, *p* < 0.0001). The *Weekday* cluster was characterized by a bi-modal pattern, with minor peaks in activity earlier in the day and later in the afternoon. The *Weekend* cluster, on the other hand, was characterized by a more even distribution of activity during waking hours.

Finally, the tests of significance comparing the clusters based on demographic variables indicated no significant differences with respect to age (*p* = 0.411), sex assigned at birth (*p* = 0.411), gender (*p* = 0.403), sexual orientation (*p* = 0.853), race (*p* = 0.162), ethnicity (*p* = 0.386), education levels (*p* = 0.932), household income (*p* = 0.161), or employment status (*p* = 0.425). The distribution of the overall sample sleep scores indicated that all participants fell within the range of moderate clinical insomnia (ISI mean = 16.52, SD = 5.27, Range = 9–27) and more than half reported poor sleep quality (PSQI mean = 7.18 SD = 2.82, Range = 3–12). While there was a trend where the lower PA was associated with slightly worse sleep scores, the p-values from the linear mixed-effects models did not indicate significant differences across the clusters (i.e., sleep disturbance—*p* = 0.0960, *p* = 0.714; ISI—*p* = 0.551, *p* = 0.700; PSQI—*p* = 0.374, *p* = 0.384).

## Discussion

This study investigated distinct latent subtypes (clusters) of accelerometer-based daily PA trajectories in an ethnically and racially diverse sample of healthy SGM adults living in the U.S. We implemented a clustering approach that uniquely accommodates hierarchical data via co-clustering at two dimensions (i.e., individuals/rows, days/columns). Our primary goal in using this technique was to allow consideration of the multiple days of data from each individual in the clustering process instead of having to aggregate the data at the person level. In addition, the functional mixture modeling framework relies on entire data curves as the unit of analysis, therefore considering the complete PA trajectories that unfold over time to provide information at the individual- and day- level. FLBMs have been implemented by others to investigate evolution of COVID cases [62] and consumer electric consumption [30]. This study therefore represents a novel application of FLBM in this health behavior context, providing new insights into PA patterns that can help inform future strategies for improving PA among SGM adults.

### Individual-level clusters

Our analyses indicated 3 distinct clusters of PA patterns at the individual level: the ‘*Low activity*’, ‘*Steady moderate activity’*, ‘*Fluctuating high activity’*. These clusters were distinguished by variations in both the trend of PA as well as the total PA volume throughout the day. For example, the *Low activity* cluster membership was associated with the least amount of activity based on all the PA outcomes estimated for this cluster. This cluster was the least likely to accumulate any daily MVPA (median MVPA = 0 min/day), suggesting the highest risk for adverse health outcomes associated with not meeting the PA guidelines. The *Low activity* cluster was also associated with the least intraday variability, further suggesting that this inactivity pattern is consistent. In comparison, the *Steady moderate activity* cluster was associated with later activity peak times compared to the *Fluctuating high activity* pattern, along with a more uniform distribution and reduced variability throughout the day. These two clusters could represent the activity profile of an adult with a typical working (e.g., 9-to-5) schedule, where some of the activity is accumulated from commuting to and from work, for example. The *Fluctuating high activity* cluster could also be representative of early risers—who wake up earlier and prefer to do activities earlier in the day. The *Steady moderate activity* cluster had relatively lower levels of activity during the day, particularly in the morning, with activity levels reaching a peak in the afternoon. There could be numerous explanations for this pattern (e.g., commuting differences, family roles, exercise habits, etc.), though we do not have additional contextual information to determine this for each participant.

Our findings differ to those of others investigating habitual PA patterns based on clustering and comparative cohort analyses. For example, a previous study [63] investigating potential latent PA profiles based on accelerometry (*N* = 7,931) indicated six clusters based on total daily volume of PA. The majority of the participants in their “most active” cluster were more likely to be younger (ages 18–34) and of Hispanic ethnicity, and to accumulate activity during work and commuting. In contrast, participants in their “least active” cluster were more likely to be older adults (ages ≥ 65), and Non-Hispanic White [63]. Our sample included mostly Hispanic adults and a relatively smaller proportion of participants were assigned to the *Low activity* cluster. However, our study did not find significant differences between the clusters with respect to age, which is consistent with other studies indicating that sedentary behavior was not higher among older participants [64]. Nevertheless, we had a smaller sample of participants, which might explain the fewer clusters we identified than those in the study by Evenson et al. [63]. With respect to previous studies focusing on PA patterns among SGM adults, our findings contribute to the currently mixed literature. For example, some studies suggest that SGM adults engage in less PA compared to their cisgender heterosexual counterparts [16, 65, 66], while others report the opposite trend [10, 67, 68]. The heterogeneity across these studies of PA in SGM people could in part be due to the use of cross-sectional and recall-based self-reported PA measures. In contrast, our sample had up to 30 days of objectively-estimated, continuous PA data for analyses. Alternatively, the specific PA variables (e.g., step counts vs. MVPA minutes vs. total activity minutes, etc.) might explain the different findings. We used step count as the clustering model outcome because it is the most commonly reported PA variable in the literature [69–71], and is intuitive, which facilitates interpretability and comparison with prior studies.

### Day-level clusters

The FLBM estimated 2 distinct, latent clusters based on the day-level data *(‘Weekday”* and *“Weekend”* clusters; see Fig. 3). The main distinction between the 2 clusters was that Saturdays, Sundays, and to a lesser extent Fridays, were more likely to be accumulated within the “*Weekend*” cluster vs. the “*Weekday”* cluster. This would suggest that on average (i.e., across the entire sample), there are differences in activity patterns on what could be seen as the typical work days vs. weekend days, which is not surprising. Overall, these results suggest within-individual stability over time in day-to-day activity levels over the course of a month, which has implications for future studies regarding the minimum required number of days to capture habitual PA patterns in this population. Nevertheless, these are secondary results and beyond the scope of our primary a priori hypotheses for this study, thus should be interpreted with caution. Future research could focus on further elucidating sample-level potential seasonal variations.

### Implications

Identifying individuals that share similar patterns (i.e., clusters) can be beneficial when designing interventions as this can help tailor the intervention according to the features of the identified clusters. The FLBM approach enables consideration of potential within-participant variations in PA as well as its temporality, not just total daily steps or PA minutes but also their intra-day distribution. Using phenotypes might also help to identify some of the barriers faced by SGM individuals that might otherwise be challenging to identify. By identifying clusters of PA, healthcare providers can also design tailored just-in-time adaptive interventions (JITAIs), such as sending reminders during long sitting periods or other times when the individual has the ability to engage in PA (e.g., proximity to a facility, break from work, or being in an LGBTQ+ -inclusive space where they feel comfortable exercising). These adaptive interventions are delivered according to specific times of day or week aligned with individuals’ lifestyles, as well as personal environmental factors, which can increase efficacy in PA engagement for individuals facing higher health risks and unique barriers to exercise, including SGM individuals who may encounter discrimination or lack of access to inclusive exercise spaces.

### Strengths

In addition to the strengths of the FLBM approach described earlier, use of 30 days of objectively-estimated PA data from trackers has the advantages of avoiding recall bias and providing more granular information. Consumer-grade wrist worn trackers (e.g., Fitbit) are easy to use, small, and typically more comfortable and attractive to wear for long periods of time, compared to research-grade accelerometers. They also have user interfaces via Apps that show activity summaries to participants in real time, which can provide additional motivation to the participant for wearing them, therefore improving wear time. Third, these devices allow data collection over extended periods of time as they can be charged by the participant, in contrast to research-grade devices that typically require specialized charging equipment by the research team and thus increasing testing and resource burden.

### Limitations

We recognize some limitations of our study. First, despite the above-mentioned advantages of consumer-grade devices, they can also be less precise than research-grade devices for PA estimation. For example, there have been reports of both underestimation [72, 73] and overestimation [73, 74] of step counts during light PA. However, others reported comparable accuracy to research-grade accelerometers [75–77], and adequate reliability for step count estimation [78–81]. Second, we did not have information on the participants’ daily activities (e.g., work-related, leisure, and household activities) to contextualize the PA patterns. Next, while 1,209 person-level days provides a sufficient analytic sample for the clustering analysis, we had a relatively small sample of 42 SGM adults living in suburban and urban areas in the U.S. As such, our results may not be generalizable to other demographics and different settings. We note that the a priori study focus included identification of PA clusters, rather than examining associations with health outcomes. Participants were screened for and excluded if they had hypertension, cardiovascular disease, sleep disorders, or cognitive impairments. As a result, our sample consisted of relatively healthy individuals, limiting our ability to explore relationships between PA and chronic disease outcomes. Combined with the small sample size, this might have reduced the likelihood of detecting significant differences in the demographic factors. Future research should examine these associations in populations with existing health conditions to further understand the implications of PA patterns in diverse SGM subgroups, with focus on sleep outcomes.

## Conclusions

Our study identified 3 latent profiles (clusters) of individuals associated with low, steady moderate, and high fluctuating PA patterns throughout the day, using 30 days of data from a racially/ethnically diverse sample of SGM adults living in a large urban city in the U.S. Identifying SGM subgroups with similar PA patterns can inform the design of tailored interventions for improving PA engagement, such as delivering prompts and nudges at specific times of the day (i.e., JITAIs). These innovative approaches also hold significant potential in addressing the unique barriers and motivations that characterize SGM individuals’ PA engagement, ultimately promoting healthier behaviors and overall well-being. Future research should test whether the identified clusters can be applied to other populations and sub-groups living in urban, suburban, and rural areas, and the presence of potential seasonal variations.

## Supplementary Information

Supplementary Material 1. 

## Data Availability

The study data are not openly available due to reasons of sensitivity and are available from the funding PI (Caceres) upon reasonable request. Data are located in controlled access data storage at Columbia University School of Nursing.

## Abbreviations

BIC: Bayesian information criterion
CDC: Centers for Disease Control and Prevention
EMAs: Ecological momentary assessments
FLBM: Functional latent block models
FMI: Fraction of missing information
ICL: Integrated completed likelihood
JITAIs: Just-in-time adaptive interventions
MICE: Multivariate imputation by chained equations
MVPA: Moderate-to-vigorous physical activity
PA: Physical activity
PMM: Predictive mean matching
RIV: Relative information variance
SGM: Sexual and gender minority
U.S.: United States
*X*^*2*^: Chi squared
