# Supplementary material for: Characterization and clustering of intra-day physical activity patterns using accelerometry among sexual and gender minority adults
Source: BMC Public Health. 2025 Jul 3;25:2294. doi: 10.1186/s12889-025-23425-5 (PMC12224577; doi:10.1186/s12889-025-23425-5)
Supplement: Supplementary file 1 — Supplementary Material 1. [file 12889_2025_23425_MOESM1_ESM.pdf]

Exit Interview

Participant ID ______________________

We will now start your final interview for the study. Please answer the questions as honestly as you can. Remember that you can skip or refuse to answer any question.

For this interview, I am going to ask you to refer to the handcard document that we previously emailed to you. This document will help you answer the questions in this interview. I will specify which handcard you should use for a particular question or set of questions.

You should answer the question by saying only the number or letter associated with the response(s) you have chosen.

Systolic BP #1 ______________________

Diastolic BP #1 ______________________

Systolic BP #2 ______________________

Diastolic BP #2 ______________________

The following questions relate to your usual sleep habits during the past month only. Your answers should indicate the most accurate reply for the majority of days and nights in the past month.

When have you usually gone to bed? (HH:MM) ______________________

When have you usually gone to bed? (AM/PM)
- ◯ AM
- ◯ PM

How long (in minutes) has it taken you to fall asleep each night? ______________________

What time have you usually gotten up in the morning? (HH:MM) ______________________

What time have you usually gotten up in the morning? (AM/PM)
- ◯ AM
- ◯ PM

How many hours of actual sleep did you get at night? ______________________

How many hours were you in bed? ______________________

Please refer to handcard #1 to answer the next set of questions.

During the past month, how often have you had trouble sleeping because you cannot get to sleep within 30 minutes

- ◯ 1--Not during the past month
- ◯ 2--Less than once a week
- ◯ 3--Once or twice a week
- ◯ 4--Three or more times a week
- ◯ DON'T KNOW
- ◯ REFUSED
- ◯ NO APPLICABLE RESPONSE

During the past month, how often have you had trouble sleeping because you wake up in the middle of the night or early morning

- ◯ 1--Not during the past month
- ◯ 2--Less than once a week
- ◯ 3--Once or twice a week
- ◯ 4--Three or more times a week
- ◯ DON'T KNOW
- ◯ REFUSED
- ◯ NO APPLICABLE RESPONSE

During the past month, how often have you had trouble sleeping because you have to get up to use the bathroom

- ◯ 1--Not during the past month
- ◯ 2--Less than once a week
- ◯ 3--Once or twice a week
- ◯ 4--Three or more times a week
- ◯ DON'T KNOW
- ◯ REFUSED
- ◯ NO APPLICABLE RESPONSE

During the past month, how often have you had trouble sleeping because you cannot breathe comfortably

- ◯ 1--Not during the past month
- ◯ 2--Less than once a week
- ◯ 3--Once or twice a week
- ◯ 4--Three or more times a week
- ◯ DON'T KNOW
- ◯ REFUSED
- ◯ NO APPLICABLE RESPONSE

During the past month, how often have you had trouble sleeping because you cough or snore loudly

- ◯ 1--Not during the past month
- ◯ 2--Less than once a week
- ◯ 3--Once or twice a week
- ◯ 4--Three or more times a week
- ◯ DON'T KNOW
- ◯ REFUSED
- ◯ NO APPLICABLE RESPONSE

During the past month, how often have you had trouble sleeping because you feel too cold

- ◯ 1--Not during the past month
- ◯ 2--Less than once a week
- ◯ 3--Once or twice a week
- ◯ 4--Three or more times a week
- ◯ DON'T KNOW
- ◯ REFUSED
- ◯ NO APPLICABLE RESPONSE

During the past month, how often have you had trouble sleeping because you feel too hot

- ◯ 1--Not during the past month
- ◯ 2--Less than once a week
- ◯ 3--Once or twice a week
- ◯ 4--Three or more times a week
- ◯ DON'T KNOW
- ◯ REFUSED
- ◯ NO APPLICABLE RESPONSE

During the past month, how often have you had trouble sleeping because you have bad dreams	◯ 1--Not during the past month<br>◯ 2--Less than once a week<br>◯ 3--Once or twice a week<br>◯ 4--Three or more times a week<br>◯ DON'T KNOW<br>◯ REFUSED<br>◯ NO APPLICABLE RESPONSE
During the past month, how often have you had trouble sleeping because you have pain	◯ 1--Not during the past month<br>◯ 2--Less than once a week<br>◯ 3--Once or twice a week<br>◯ 4--Three or more times a week<br>◯ DON'T KNOW<br>◯ REFUSED<br>◯ NO APPLICABLE RESPONSE
Other reason(s), please describe, including how often you have had trouble sleeping because of this reason(s):	______________________________
If no reason given assign participant score of "Not in the past month (0)"<br><br>OTHERWISE: Please describe how often you have had trouble sleeping because of this reason(s)?	◯ 1--Not during the past month<br>◯ 2--Less than once a week<br>◯ 3--Once or twice a week<br>◯ 4--Three or more times a week<br>◯ DON'T KNOW<br>◯ REFUSED<br>◯ NO APPLICABLE RESPONSE
During the past month, how often have you taken medicine (prescribed or "over the counter") to help you sleep?	◯ 1--Not during the past month<br>◯ 2--Less than once a week<br>◯ 3--Once or twice a week<br>◯ 4--Three or more times a week<br>◯ DON'T KNOW<br>◯ REFUSED<br>◯ NO APPLICABLE RESPONSE
During the past month, how often have you had trouble staying awake while driving, eating meals, or engaging in social activity?	◯ 1--Not during the past month<br>◯ 2--Less than once a week<br>◯ 3--Once or twice a week<br>◯ 4--Three or more times a week<br>◯ DON'T KNOW<br>◯ REFUSED<br>◯ NO APPLICABLE RESPONSE
During the past month, how much of a problem has it been for you to keep up enthusiasm to get things done?	◯ 1--Not during the past month<br>◯ 2--Less than once a week<br>◯ 3--Once or twice a week<br>◯ 4--Three or more times a week<br>◯ DON'T KNOW<br>◯ REFUSED<br>◯ NO APPLICABLE RESPONSE
During the past month, how would you rate your sleep quality overall?	◯ Very good<br>◯ Fairly good<br>◯ Fairly bad<br>◯ Very bad<br>◯ DON'T KNOW<br>◯ REFUSED<br>◯ NO APPLICABLE RESPONSE

PROMIS-Sleep Disturbance

Please respond to each question or statement by selecting one choice pertaining to sleep disturbance in the past 7 days.

In the past 7 days, my sleep quality was	◯ Very good<br>◯ Good<br>◯ Fair<br>◯ Poor<br>◯ Very poor<br>◯ DON'T KNOW<br>◯ REFUSED<br>◯ NO APPLICABLE RESPONSE

(Please refer to handcard #2 for the next few questions.)

In the past 7 days, my sleep was refreshing	◯ 1--Very much<br>◯ 2--Quite a bit<br>◯ 3--Somewhat<br>◯ 4--A little bit<br>◯ 5--Not at all<br>◯ DON'T KNOW<br>◯ REFUSED<br>◯ NO APPLICABLE RESPONSE
In the past 7 days, I had a problem with my sleep	◯ 1--Very much<br>◯ 2--Quite a bit<br>◯ 3--Somewhat<br>◯ 4--A little bit<br>◯ 5--Not at all<br>◯ DON'T KNOW<br>◯ REFUSED<br>◯ NO APPLICABLE RESPONSE
In the past 7 days, I had difficulty falling asleep	◯ 1--Very much<br>◯ 2--Quite a bit<br>◯ 3--Somewhat<br>◯ 4--A little bit<br>◯ 5--Not at all<br>◯ DON'T KNOW<br>◯ REFUSED<br>◯ NO APPLICABLE RESPONSE
In the past 7 days, my sleep was restless	◯ 1--Very much<br>◯ 2--Quite a bit<br>◯ 3--Somewhat<br>◯ 4--A little bit<br>◯ 5--Not at all<br>◯ DON'T KNOW<br>◯ REFUSED<br>◯ NO APPLICABLE RESPONSE

In the past 7 days, I tried hard to get to sleep

- ◯ 1--Very much
- ◯ 2--Quite a bit
- ◯ 3--Somewhat
- ◯ 4--A little bit
- ◯ 5--Not at all
- ◯ DON'T KNOW
- ◯ REFUSED
- ◯ NO APPLICABLE RESPONSE

In the past 7 days, I worried about not being able to fall asleep

- ◯ 1--Very much
- ◯ 2--Quite a bit
- ◯ 3--Somewhat
- ◯ 4--A little bit
- ◯ 5--Not at all
- ◯ DON'T KNOW
- ◯ REFUSED
- ◯ NO APPLICABLE RESPONSE

In the past 7 days, I was satisfied with my sleep

- ◯ 1--Very much
- ◯ 2--Quite a bit
- ◯ 3--Somewhat
- ◯ 4--A little bit
- ◯ 5--Not at all
- ◯ DON'T KNOW
- ◯ REFUSED
- ◯ NO APPLICABLE RESPONSE

Please refer to handcard #3 for the next few questions.

Please respond to each item by selecting one choice per question about your sleep in the past 7 days.

In the past 7 days, I had a hard time getting things done because I was sleepy

- ◯ 1--Not at all
- ◯ 2--A little bit
- ◯ 3--Somewhat
- ◯ 4--Quite a bit
- ◯ 5--Very much
- ◯ DON'T KNOW
- ◯ REFUSED
- ◯ NO APPLICABLE RESPONSE

In the past 7 days, I felt alert when I woke up

- ◯ 1--Not at all
- ◯ 2--A little bit
- ◯ 3--Somewhat
- ◯ 4--Quite a bit
- ◯ 5--Very much
- ◯ DON'T KNOW
- ◯ REFUSED
- ◯ NO APPLICABLE RESPONSE

In the past 7 days, I felt tired	◯ 1--Not at all<br>◯ 2--A little bit<br>◯ 3--Somewhat<br>◯ 4--Quite a bit<br>◯ 5--Very much<br>◯ DON'T KNOW<br>◯ REFUSED<br>◯ NO APPLICABLE RESPONSE
In the past 7 days, I had problems during the day because of poor sleep	◯ 1--Not at all<br>◯ 2--A little bit<br>◯ 3--Somewhat<br>◯ 4--Quite a bit<br>◯ 5--Very much<br>◯ DON'T KNOW<br>◯ REFUSED<br>◯ NO APPLICABLE RESPONSE
In the past 7 days, I had a hard time concentrating because of poor sleep	◯ 1--Not at all<br>◯ 2--A little bit<br>◯ 3--Somewhat<br>◯ 4--Quite a bit<br>◯ 5--Very much<br>◯ DON'T KNOW<br>◯ REFUSED<br>◯ NO APPLICABLE RESPONSE
In the past 7 days, I felt irritable because of poor sleep	◯ 1--Not at all<br>◯ 2--A little bit<br>◯ 3--Somewhat<br>◯ 4--Quite a bit<br>◯ 5--Very much<br>◯ DON'T KNOW<br>◯ REFUSED<br>◯ NO APPLICABLE RESPONSE
In the past 7 days, I was sleepy during the daytime	◯ 1--Not at all<br>◯ 2--A little bit<br>◯ 3--Somewhat<br>◯ 4--Quite a bit<br>◯ 5--Very much<br>◯ DON'T KNOW<br>◯ REFUSED<br>◯ NO APPLICABLE RESPONSE
In the past 7 days, I had trouble staying awake during the day	◯ 1--Not at all<br>◯ 2--A little bit<br>◯ 3--Somewhat<br>◯ 4--Quite a bit<br>◯ 5--Very much<br>◯ DON'T KNOW<br>◯ REFUSED<br>◯ NO APPLICABLE RESPONSE

The following questions ask about your experiences participating in this study over the past month. The information you provide will help us plan future studies related to health in LGBTQ+ adults.

(Please refer to handcard #4 for the next few questions.)

How would you rate your overall experience participating in this study?	◯ 1--Poor<br>◯ 2--Fair<br>◯ 3--Good<br>◯ 4--Very good<br>◯ 5--Excellent<br>◯ DON'T KNOW<br>◯ REFUSED<br>◯ NO APPLICABLE RESPONSE
How would you rate your experience answering the questions about your health during the first visit?	◯ 1--Poor<br>◯ 2--Fair<br>◯ 3--Good<br>◯ 4--Very good<br>◯ 5--Excellent<br>◯ DON'T KNOW<br>◯ REFUSED<br>◯ NO APPLICABLE RESPONSE
How would you rate your experience giving the saliva samples?	◯ 1--Poor<br>◯ 2--Fair<br>◯ 3--Good<br>◯ 4--Very good<br>◯ 5--Excellent<br>◯ DON'T KNOW<br>◯ REFUSED<br>◯ NO APPLICABLE RESPONSE
How would you rate the experience of your saliva sample being used for DNA analysis (if applicable)?	◯ 1--Poor<br>◯ 2--Fair<br>◯ 3--Good<br>◯ 4--Very good<br>◯ 5--Excellent<br>◯ DON'T KNOW<br>◯ REFUSED<br>◯ NO APPLICABLE RESPONSE
How would you rate your experience wearing the Fitbit watch for 30 days?	◯ 1--Poor<br>◯ 2--Fair<br>◯ 3--Good<br>◯ 4--Very good<br>◯ 5--Excellent<br>◯ DON'T KNOW<br>◯ REFUSED<br>◯ NO APPLICABLE RESPONSE
How would you rate your experience taking your own blood pressure twice a day for 30 days?	◯ 1--Poor<br>◯ 2--Fair<br>◯ 3--Good<br>◯ 4--Very good<br>◯ 5--Excellent<br>◯ DON'T KNOW<br>◯ REFUSED<br>◯ NO APPLICABLE RESPONSE

How would you rate your experience completing the daily surveys in this study?

◯ 1--Poor
◯ 2--Fair
◯ 3--Good
◯ 4--Very good
◯ 5--Excellent
◯ DON'T KNOW
◯ REFUSED
◯ NO APPLICABLE RESPONSE

How would you rate your experience interacting with the study staff?

◯ 1--Poor
◯ 2--Fair
◯ 3--Good
◯ 4--Very good
◯ 5--Excellent
◯ DON'T KNOW
◯ REFUSED
◯ NO APPLICABLE RESPONSE

The following questions ask about your impressions about the activities we asked you to complete in this study and if you would participate in a similar study in the future.

(Please refer to handcard #5 for the next few questions.)

Did you think the length of the morning survey you completed each day was:

◯ 1--Too short
◯ 2--The right length
◯ 3--Too long
◯ DON'T KNOW
◯ REFUSED
◯ NO APPLICABLE RESPONSE

Did you think the length of the evening survey you completed each day was:

◯ 1--Too short
◯ 2--The right length
◯ 3--Too long
◯ DON'T KNOW
◯ REFUSED
◯ NO APPLICABLE RESPONSE

Was 30 days of daily surveys:

◯ 1--Too short
◯ 2--The right length
◯ 3--Too long
◯ DON'T KNOW
◯ REFUSED
◯ NO APPLICABLE RESPONSE

Was 30 days of blood pressure measurement...

◯ 1--Too short
◯ 2--The right length
◯ 3--Too long
◯ DON'T KNOW
◯ REFUSED
◯ NO APPLICABLE RESPONSE

Was 30 days of wearing a Fitbit watch...

◯ 1--Too short
◯ 2--The right length
◯ 3--Too long
◯ DON'T KNOW
◯ REFUSED
◯ NO APPLICABLE RESPONSE

(Please refer to handcard #6 for the next few questions.)

The Fitbit watch interfered with my sleep...	◯ 1--Strongly disagree<br>◯ 2--Disagree<br>◯ 3--Somewhat disagree<br>◯ 4--Neither agree nor disagree<br>◯ 5--Somewhat agree<br>◯ 6--Agree<br>◯ 7--Strongly agree<br>◯ DON'T KNOW<br>◯ REFUSED<br>◯ NO APPLICABLE RESPONSE
I would participate in a similar study with the same home blood pressure monitoring for 30 days...	◯ 1--Strongly disagree<br>◯ 2--Disagree<br>◯ 3--Somewhat disagree<br>◯ 4--Neither agree nor disagree<br>◯ 5--Somewhat agree<br>◯ 6--Agree<br>◯ 7--Strongly agree<br>◯ DON'T KNOW<br>◯ REFUSED<br>◯ NO APPLICABLE RESPONSE
I would participate in a similar study with the same home blood pressure monitoring for 14 days (two weeks)...	◯ 1--Strongly disagree<br>◯ 2--Disagree<br>◯ 3--Somewhat disagree<br>◯ 4--Neither agree nor disagree<br>◯ 5--Somewhat agree<br>◯ 6--Agree<br>◯ 7--Strongly agree<br>◯ DON'T KNOW<br>◯ REFUSED<br>◯ NO APPLICABLE RESPONSE
I would participate in a similar study that uses daily surveys for 30 days...	◯ 1--Strongly disagree<br>◯ 2--Disagree<br>◯ 3--Somewhat disagree<br>◯ 4--Neither agree nor disagree<br>◯ 5--Somewhat agree<br>◯ 6--Agree<br>◯ 7--Strongly agree<br>◯ DON'T KNOW<br>◯ REFUSED<br>◯ NO APPLICABLE RESPONSE
I would participate in a similar study that uses daily surveys for 14 days (two weeks)...	◯ 1--Strongly disagree<br>◯ 2--Disagree<br>◯ 3--Somewhat disagree<br>◯ 4--Neither agree nor disagree<br>◯ 5--Somewhat agree<br>◯ 6--Agree<br>◯ 7--Strongly agree<br>◯ DON'T KNOW<br>◯ REFUSED<br>◯ NO APPLICABLE RESPONSE

I would participate in a similar study that requires me to wear a Fitbit watch for 30 days...	◯ 1--Strongly disagree<br>◯ 2--Disagree<br>◯ 3--Somewhat disagree<br>◯ 4--Neither agree nor disagree<br>◯ 5--Somewhat agree<br>◯ 6--Agree<br>◯ 7--Strongly agree<br>◯ DON'T KNOW<br>◯ REFUSED<br>◯ NO APPLICABLE RESPONSE
I would participate in a similar study that requires me to wear a Fitbit watch for 14 days (two weeks)...	◯ 1--Strongly disagree<br>◯ 2--Disagree<br>◯ 3--Somewhat disagree<br>◯ 4--Neither agree nor disagree<br>◯ 5--Somewhat agree<br>◯ 6--Agree<br>◯ 7--Strongly agree<br>◯ DON'T KNOW<br>◯ REFUSED<br>◯ NO APPLICABLE RESPONSE
I would participate in a similar study that includes collection of two saliva samples collected in the same manner as this study.	◯ 1--Strongly disagree<br>◯ 2--Disagree<br>◯ 3--Somewhat disagree<br>◯ 4--Neither agree nor disagree<br>◯ 5--Somewhat agree<br>◯ 6--Agree<br>◯ 7--Strongly agree<br>◯ DON'T KNOW<br>◯ REFUSED<br>◯ NO APPLICABLE RESPONSE
I would participate in a similar study that includes collection of a small sample of blood.	◯ 1--Strongly disagree<br>◯ 2--Disagree<br>◯ 3--Somewhat disagree<br>◯ 4--Neither agree nor disagree<br>◯ 5--Somewhat agree<br>◯ 6--Agree<br>◯ 7--Strongly agree<br>◯ DON'T KNOW<br>◯ REFUSED<br>◯ NO APPLICABLE RESPONSE
How would you rate your experience completing the daily surveys in this study?	◯ Poor<br>◯ Fair<br>◯ Good<br>◯ Very Good<br>◯ Excellent<br>◯ DON'T KNOW<br>◯ REFUSED<br>◯ NO APPLICABLE RESPONSE

Lastly, we are interested in your honest opinion about participating in this study. The next three questions will help us determine how we might possibly change the study in the future.

What did you like best about participating in this study? __________________________________

What did you like least about participating in this study? __________________________________

Do you have any other feedback you'd like to give us about this study? __________________________________
